# Knowledge and practice of immediate newborn care among midwives and nurses in public health facilities of Afar regional state, Northeast Ethiopia

**DOI:** 10.1186/s12884-019-2581-3

**Published:** 2019-11-19

**Authors:** Hawa Abdu, Measho Gebrselassie, Mohammed Abdu, Kusse Urmale Mare, Woldemichael Tadesse, Misgan Legesse Liben

**Affiliations:** 1 Family Health Case Team Coordinator, Regional Health Bureau, Samara, Afar Ethiopia; 2 School of Public Health, College of Health Sciences, Mekelle University, Mekelle, Tigray Ethiopia; 3 Department of Midwifery, College of Medical and Health Sciences, Samara University, Samara, Afar Ethiopia; 4 Department of Nursing, College of Medical and Health Sciences, Samara University, Samara, Afar Ethiopia; 5 Department of Public Health, College of Health Sciences, Woldia University, Woldia, Amhara Ethiopia

**Keywords:** Afar, Immediate, Knowledge, Practice, Newborn care

## Abstract

**Background:**

The care given to newborns immediately within the first few hours of birth is critical for their survival. However, their survival depends on the health professional’s knowledge and skills to deliver appropriate newborn care interventions. Therefore, this study aimed to assess the knowledge and practice of immediate newborn care among nurses and midwives in public health facilities of Afar Regional State, Northeast Ethiopia.

**Methods:**

Institution based cross-sectional study design was employed on 357 nurses and midwives working in 48 public health facilities (45 health centers and 3 hospitals) during April 2018. Data were collected using interviewer-administered questionnaire and observation checklist. Then, data were entered into Epi-info version 7.0 and exported to SPSS version 20 for analysis. Univariable and multivariable logistic regression analyses were carried out to estimate odds ratio with 95% confidence interval. A *p*-value less than 0.05 was used to declare statistical significance.

**Results:**

Overall, 53.8% [95% CI: (48.6, 59.0%)] and 62.7% [(95% CI: (57.7, 67.8%))] of the health professionals (midwives and nurses) had adequate knowledge and good practice on immediate newborn care, respectively. Working in hospital [AOR: 4.62; 95% CI (1.76, 12.10)], being a female [AOR: 0.59; 95% CI (0.39, 0.98)] and interested in providing newborn care [AOR: 0.29; 95% CI (0.13, 0.68)] were positively associated with having adequate knowledge on immediate newborn care. On the other hand, having work experience of < 5 years [AOR: 0.33; 95% CI (0.14, 0.78)], inadequate knowledge [AOR: 0.39; 95% CI (0.25, 0.64)], having work load [AOR: 2.09; 95% CI (1.17, 3.73)], being not interested to provide immediate newborn care [AOR: 0.35; 95% CI (0.16, 0.74)] and working in health center [AOR: 8.56; 95% CI (2.39, 30.63)] were negatively associated with good immediate newborn care practices.

**Conclusions:**

A significant number of nurses and midwives had inadequate knowledge and poor practice on immediate newborn care. Therefore, providing a comprehensive newborn care training and creating an opportunity for nurses and midwives working at health centers to share experience from those hired in hospitals are very crucial to improve their knowledge and skills on newborn care.

## Background

A newborn is an infant who is within hours, days, or up to a few weeks from birth or it refers to an infant in the first 28 days of life [1–4]. The day of birth is the riskiest time to a baby. Newborns are very vulnerable to disease in the first week of life [1–4], where large numbers of children die soon after birth [5]. An infant is about 500 times more likely to die on the first day of life than at one month of age [2].

According to the World Health Organization (WHO) report on 2015, globally 2.7 million neonates die in the first 28 days which constitutes 45% of the under-five mortality and nearly 58% of infant mortality. This is about 75% of the neonatal mortality in the first week of birth [6, 7]. Most neonatal deaths are in low and middle-income countries [8]. In Sub-Saharan Africa, one in eleven children dies before the age of five years. This is nearly 15 times higher compared to the rate in developed countries. Furthermore, in 2013, 6.3 million children died in Sub-Saharan Africa and Southeast Asia [2, 9, 10]. If the trend continues like this, the share of neonatal deaths to under-five death is projected to increase from 45% in 2015 to 52% in 2030 [7].

In Ethiopia, where childhood mortality is higher in rural areas than in urban areas,1 in 17 children dies before the first birthday, and 1 from 11 children before the fifth birthday. The neonatal mortality and post-neonatal mortality rate were 37 and 22 deaths per 1000 live births, respectively [11]. According to the 2016 Ethiopia Demographic and Health Survey (EDHS) report, the under-5 mortality rate in Afar region was 125 deaths per 1000 live births. This is approximately two folds of the national figure of 67 deaths per 1000 live births [12].

The care given to newborns in the transitional period (immediately after birth) is crucial to their survival. Newborn care in the immediate post-delivery period includes prevention and management of hemorrhage, thermal care, cord care, early initiation of breastfeeding, eye care and recognition of when to refer. To care for newborns, nurses and midwives require knowledge and skills to provide immediate newborn care interventions [1, 3, 5].

Ethiopia has implemented multiple high impact interventions to tackle the bottlenecks of safe childhood services like inadequate care at health facilities [12, 13]. However, attendance of skilled health workers during delivery (16.4%) and postnatal care (13%) is still very low. Majority of mothers deliver at home in the presence of traditional birth attendants, which has resulted in many harmful traditional practices on newborns. This resulted in a high rate of neonatal morbidity and mortality in the first 24 h of life [14]. Moreover, globally, in spite of the provision of in-service training on immediate newborn care to most of the health professionals (93%), nearly half of the health professionals (50.25%) had poor knowledge [15].

The knowledge and practice of health care workers on immediate newborn care are vital to reduce neonatal morbidity and mortality. A study in Egypt revealed that nurses had inadequate knowledge and poor practice on immediate newborn cares [16]. However, studies in India found that 41% of health care providers [17] and 40% of nurses [18] had adequate knowledge about newborn care. Moreover, in Pune city, 72 and 98% of nurses had average knowledge and good practice on providing immediate newborn care respectively [19]. According to a study in Uganda, about 47% of health care professionals had adequate knowledge on newborn care [20]. Likewise, cross-sectional studies in Ethiopia revealed that 75% [21] and 56% [22] of health professionals had adequate knowledge about the care given to newborns immediately after birth. It was also reported that 73% [21] and 60% [22] of them had a good practice on immediate newborn care.

In Ethiopia in general, Afar regional state in particular, there is no clear evidence on the knowledge and practice of midwives and nurses towards immediate newborn care. Therefore, this study aimed to assess the knowledge and practice of immediate newborn care among midwives and nurses in public health facilities of Afar Regional State, Northeast Ethiopia.

## Methods

### Study setting and period

An institution-based cross-sectional study was conducted during April 2018 on midwives and nurses working in government health facilities in Zone one and Zone three of Afar Regional State, Northeast Ethiopia. In Zone one, there are 27 health centers and 2 hospitals, while in Zone three, there are 18 health centers and 1 hospital. In these two zones, there are 287 midwives and 326 nurses (613 nurses and midwives).

### Sample size and sampling procedure

A sample size of 386 was determined using the following formula;

$$ \mathrm{n}=\left[\frac{{\left(\mathrm{z}\frac{\upalpha}{2}\right)}^2\mathrm{p}\left(1-\mathrm{p}\right)}{{\mathrm{d}}^2}\right] $$

**Assumptions:** *n* = required sample size, $\mathrm{z}\frac{\upalpha}{2}$ = critical value for normal distribution at 95% confidence level (1.96), *P* = 60% proportion of good immediate newborn care practice among health professionals [22], d = 0.05 (margin of error), and 5% for non-response.

There are five zones in the Afar regional state. Using the rule of thumb, two zones (Zone one and three) were randomly selected. All 48 public health facilities (45 health centers and 3 hospitals) found in the selected zones were included in the study. Then, the sample size was proportionally allocated for each health facility based on the number of nurses and midwives assigned on newborn care (delivery care) unit. Since the number of nurses and midwives in each of the health facility is not equal, the sample size allocated for each facility was proportionally allocated to determine the number of nurses and midwives included in the study from each facility. Finally, all randomly selected midwives and nurses who were providing immediate newborn care during data collection period were included in the study.

### Data collection instruments and procedures

Data were collected using interviewer administered questionnaire and observation checklist. The questionnaire and observation checklist was adopted and modified from WHO and other related sources [11, 13, 23, 24]. The questionnaire was organized in three parts; socio-demographic characteristics, knowledge and practice of midwives and nurses on immediate newborn care. Data were collected by six public health professionals using the English version of the questionnaire and observation checklist. Data collectors and supervisors were trained for three days on the study instrument and data collection procedures. A pretest was conducted on 5% of the sample size in Zone two of Afar Regional State. Then, the questionnaire was improved and contextualized to fit the local condition and the study objective.

### Study variables

The dependent (outcome) variables for this study were the level of knowledge on immediate newborn care and immediate newborn care practice. Level of knowledge was assessed using 10 major questions. Nurses and midwives were considered as having adequate knowledge (coded as 1) if they respond to greater than or equal to the mean score (5.54) of the 10 knowledge-related questions. If they respond to less than the mean score of knowledge questions, considered as having inadequate knowledge (coded as 0). Good practice (coded as 1): if nurses and midwives correctly performed greater than or equal to the mean score (16.92) of the 23 practice-related questions. Poor practice (coded as 0): if they performed less than the mean score of the 23 practice questions. The independent variables were: age, sex, religion, marital status, ethnicity, educational level, monthly income, work environment (the type of health facility), experience, participation on immediate newborn care training and workload.

### Data management and statistical analysis

Data were checked for completeness and inconsistencies. Epi-info version 7.0 was used to enter, clean and code the data. Then, SPSS version 20 was used to analyse the data. Chi-Square (χ^2^) test of independence was used to determine the association between outcome variables (knowledge and practice) and each of the predictor variables. Before conducting the multivariable logistic regression analysis, preliminary analyses were conducted to assess multicollinearity. All correlations among the independent variables were weak to moderate. This indicates that multicollinearity was unlikely to be a problem [25]. The crude odds ratio (COR) was estimated in the univariable logistic regression analysis. Variables with *p*-value < 0.25 in the univariable logistic regression analysis were included in the multivariable logistic regression analysis [26, 27]. Adjusted Odds Ratio (AOR) with 95% confidence interval was estimated to assess the strength of the association. A p-value < 0.05 was used to declare statistical significance.

### Ethical considerations

This study was approved by the Research Ethics Review Committee (RERC) of Samara University dated April 15, 2018, and numbered ERC0056/2018. Letter of the permission was secured from the Regional health bureau and all selected health institutions. Informed consent was taken from the study participants after informing the study subjects on study objectives, expected outcomes, benefits and the risks associated with it. Confidentiality of responses was maintained throughout the study.

## Results

### Socio-demographic characteristics of study participants

Totally, 357 midwives and nurses participated in the study (estimated response rate of 92.5%). About 56% of the study participants were female. The mean (±SD) age of the study participants was 29.9 (±3.4) years (data not shown). Majority of the participants 117 (66.7%) were Ethiopian Orthodox followers, and 214 (59.9%) were diploma holders. Nearly 85% of the study participants were working in a health center, and 39% received training on immediate newborn care (Table 1**).**

**Table 1:** Socio-demographic Characteristics and univariate investigation of the knowledge and practice of Nurses and Midwives on immediate newborn care in Public Health Facilities of Afar Regional State, Northeastern Ethiopia, 2018

Variables	Professional category (*n* = 357)		χ^2^ *P*-value for knowledge	χ^2^ *P*-value for practice	Total n (%)
	Midwife (*n* = 231) n (%)	Nurse (*n* = 126) n (%)			
Age					
20–24	20 (8.7)	17 (13.5)	0.40	0.11	37 (10.4)
25–29	157 (68.0)	79 (62.7)			236 (66.1)
30–34	43 (18.6)	23 (18.3)			66 (18.5)
35+	11 (4.8)	7 (5.6)			18 (5.0)
Sex of respondents					
Male	128 (55.4)	73 (57.9)	0.65	< 0.001	201 (56.3)
Female	103 (44.6)	53 (42.1)			156 (43.7)
Religion					
Muslim	75 (32.5)	42 (33.3)	0.14	< 0.001	117 (32.8)
Orthodox	154 (66.7)	84 (66.7)			238 (66.7)
Protestant	2 (0.9)	0 (0.0)			2 (0.5)
Marital Status					
Single	94 (40.7)	51 (40.5)	0.01	< 0.001	145 (40.6)
Married	132 (57.1)	74 (58.7)			206 (57.7)
Divorced	5 (2.2)	1 (0.8)			6 (1.7)
Ethnicity					
Amhara	133 (57.6)	70 (55.6)	0.01	< 0.001	203 (56.9)
Afar	39 (16.9)	22 (17.6)			61 (17.0)
Tigre	34 (14.7)	20 (15.9)			54 (15.1)
Oromo	22 (9.5)	9 (7.1)			31 (8.7)
Others*	3 (1.3)	5 (4.0)			8 (2.4)
Educational level					
Diploma	135 (58.4)	79 (62.7)	0.12	0.04	214 (59.9)
Degree	96 (41.6)	47 (37.3)			143 (40.1)
Work Environment					
Hospital	34 (14.7)	21 (16.7)	< 0.001	< 0.001	55 (15.4)
Health Center	197 (85.3)	105 (83.3)			302 (84.6)
Work Experience (in years)					
< 1	22 (9.5)	11 (8.7)	0.02	< 0.001	33 (9.2)
1–3	134 (58.0)	68 (54.0)			202 (56.6)
> 3	75 (32.5)	47 (37.3)			122 (34.2)
Have interest in providing newborn care					
No	29 (12.6)	13 (10.3)	< 0.001	< 0.001	42 (11.8)
Yes	202 (87.4)	113 (89.7)			315 (88.2)
Have Work Load					
No	74 (32.0)	41 (32.5)	0.04	0.51	115 (32.2)
Yes	157 (68.0)	85 (67.5)			242 (67.8)
Received newborn care training					
No	138 (59.7)	77 (61.1)	< 0.001	< 0.001	215 (60.2)
Yes	93 (40.3)	49 (38.9)			142 (39.8)
Frequency of newborn care trainings received (*n* = 142)					
1	65 (69.9)	30 (61.2)	0.01	< 0.001	95 (66.9)
2	16 (17.2)	16 (32.5)			32 (22.5)
3	12 (12.9)	3 (6.1)			15 (10.6)

*Gurage, Wolayita, Harar, Sidamo

### Knowledge of nurses and midwives on immediate newborn care

About two-third (60.8%) of the study subjects stated that newborn should be placed on to mother’s abdomen immediately after birth, and 53.2% of the participants knew the importance of assessing breathing of newborn. Overall, 53.8% [95% CI: 48.6, 59.0%] of the participants had adequate knowledge on immediate newborn care **(**Table 2**).**

**Table 2:** Knowledge of Nurses and Midwives on immediate newborn care and independent sample t test in Afar Regional State, Northeastern Ethiopia, 2018

Variables	Professional category n (%)		t-test *p*-value	Total n (%)
	Midwife	Nurse		
Deliver baby on to mother’s abdomen				
Yes	138 (59.7)	79 (62.7)	0.26	217 (60.8)
No	93 (40.3)	47 (37.3)		140 (39.2)
Dry baby				
Yes	111 (48.1)	60 (47.6)	0.87	171 (47.9)
No	120 (51.9)	66 (52.4)		186 (52.1)
Assess breathing				
Yes	122 (52.8)	68 (54.0)	0.66	190 (53.2)
No	109 (47.2)	58 (46.0)		167 (46.8)
Cord care				
Yes	141 (61.0)	70 (55.6)	0.08	211 (59.1)
No	90 (39.0)	56 (44.4)		146 (40.9)
Initiate breastfeeding within one hour				
Yes	114 (49.4)	55 (43.7)	0.05	169 (47.3)
No	117 (50.6)	71 (56.3)		188 (52.7)
Skin to skin contact				
Yes	105 (45.5)	51 (40.5)	0.05	156 (43.7)
No	126 (54.5)	75 (59.5)		201 (56.3)
Eye care				
Yes	144 (62.3)	80 (63.5)	0.66	224 (62.7)
No	87 (37.7)	46 (36.5)		133 (37.3)
Vitamin k injection				
Yes	145 (62.8)	72 (57.1)	0.06	217 (60.8)
No	86 (37.2)	54 (42.9)		140 (39.2)
Weighing baby				
Yes	127 (55.0)	69 (54.8)	0.94	196 (54.9)
No	104 (45.0)	57 (45.2)		161 (45.1)
Immunization				
Yes	154 (66.7)	74 (58.7)	0.01	228 (63.9)
No	77 (33.3)	52 (41.3)		129 (36.1)
Over all knowledge				
Adequate	128 (55.4)	64 (50.8)	0.24	192 (53.8)
Inadequate	103 (44.6)	62 (49.2)		165 (46.2)

### Factors affecting knowledge of nurses and midwives on immediate newborn care

The result from the univariate analysis revealed that there was an association between respondents’ background characteristics and their knowledge on immediate newborn care. Work environment and experience, interest in providing newborn care and being received newborn care training were significantly associated with nurses and midwives’ knowledge about newborn care (Table 1).

The univariable logistic regression analysis showed that age, having an interest in providingnewborn care, receiving training, workload and environment were significantly associated with the knowledge of nurses and midwives on immediate newborn care. However, the multivariable logistic regression analysis showed that the odds of having adequate knowledge about immediate newborn care was significantly higher for nurses and midwives working in hospitals [AOR: 4.62; 95% CI (1.76,12.10)] compared to those working in health centers. Male midwives and nurses had lower odds of having adequate knowledge on immediate newborn care [AOR: 0.59; 95% CI (0.39, 0.98)] compared to females. Midwives and nurses without interest to provide immediate newborn care were less likely to [AOR: 0.29; 95% CI (0.13,0.68)] have adequate knowledge on immediate newborn care compared to those who had the interest to provide (Table 3).

**Table 3:** Univariable and multivariable logistic regression analysis of Knowledge of Nurses and Midwives on immediate newborn care in public health facilities of Afar Region, Northeastern Ethiopia, 2018

Predictors	Knowledge n (%)		COR (95% CI)	AOR (95% CI)
	Adequate	Inadequate		
Age of participant				
20–24	19 (9.9)	19 (11.5)	0.20 (0.05,0.81)*	0.64 (0.17,2.46)
25–29	122 (63.5)	113 (68.5)	0.22 (0.06,0.77)*	0.63 (0.19,2.12)
30–34	36 (18.8)	30 (18.2)	0.24 (0.06,0.91)*	0.60 (0.16,2.28)
35+	15 (7.8)	3 (1.8)	1	1
Sex of respondent				
Male	102 (53.1)	99 (60.0)	0.76 (0.49,1.15)	0.59 (0.39, 0.98) *
Female	90 (46.9)	66 (40.0)	1	1
Educational level				
Diploma	109 (56.8)	105 (63.6)	0.75 (0.49,1.15)	0.91 (0.55, 1.53)
Degree	83 (43.2)	60 (36.4)	1	1
Work environment				
Hospital	52 (27.1)	3 (1.8)	20.06 (6.13,65.34)*	4.62 (1.76,12.10) *
Health center	140 (72.9)	162 (98.2)	1	1
Interest in providing newborn care				
No	5 (2.6)	37 (22.4)	0.09 (0.06,0.24)*	0.29 (0.13,0.68) *
Yes	187 (97.4)	128 (77.6)	1	1
Have Work Load				
No	49 (25.5)	66 (40.0)	0.51 (0.33,0.81)*	0.87 (0.59,1.54)
Yes	143 (74.5)	99 (60.0)	1	1
Received newborn care training				
No	89 (46.4)	126 (76.4)	0.28 (0.17,0.42)*	0.74 (0.41,1.33)
Yes	103 (53.6)	39 (23.6)	1	1
Professional category				
Midwife	103 (62.4)	128 (66.7)	1.20 (0.78,1.86)	1.33 (0.82,2.15)
Nurse	62 (37.6)	64 (33.3)	1	1

*Significant at *p* < 0.05. COR: crude odds ratio; AOR: adjusted odds ratio; CI: confidence interval

### The practice of nurses and midwives on immediate newborn care

Three hundred and thirty-one (92.7%), 230 (64.4%) and 282 (79%) of the study participants put on a sterile glove, clean the eyes of newborn and kept baby skin-to-skin contact with mother, respectively. The majority (80.7%) of the nurses and midwives applied tetracycline eye ointment for the prevention of ophthalmic neonatorum, but only 166 (46.5%) gave the recommended immunization (Oral Polio Vaccine (OPV_0_) and Bacillus Calmette-Guerin (BCG) vaccine) immediately after birth. Overall, 62.7% [95% CI: 57.7, 67.8%] of the study participants have a good practice on immediate newborn care (Table 4).

**Table 4:** Practice of Nurses and Midwives on immediate newborn care and independent sample t test in Afar Regional State, Northeastern Ethiopia, 2018

Variables	Professional category n (%)		t-test *p*-value	Total n (%)
	Midwife	Nurse		
Hand washing prior to care				
No	125 (54.1)	63 (50.0)	0.36	188 (52.7)
Yes	106 (45.9)	63 (50.0)		169 (47.3)
Put on sterile glove				
No	18 (7.8)	8 (6.3)	0.32	26 (7.3)
Yes	213 (92.2)	118 (93.7)		331 (92.7)
Wipes eye after head is delivered				
No	87 (37.7)	55 (43.7)	0.05	142 (39.8)
Yes	144 (62.3)	71 (56.3)		215 (60.2)
Clean eyes appropriately				
No	81 (35.1)	46 (36.5)	0.59	127 (35.6)
Yes	150 (64.9)	80 (63.5)		230 (64.4)
Immediately drying the baby				
No	40 (17.3)	17 (13.5)	0.05	57 (16.0)
Yes	191 (82.7)	109 (86.5)		300 (84.0)
Delivery surface sterile				
No	67 (29.0)	32 (25.4)	0.14	99 (27.7)
Yes	164 (71.0)	94 (74.6)		258 (72.3)
Remove wet cloth				
No	51 (22.1)	23 (18.3)	0.08	74 (20.7)
Yes	180 (77.9)	103 (81.7)		283 (79.3)
Skin to skin contact				
No	48 (20.8)	27 (21.4)	0.78	75 (21.0)
Yes	183 (79.2)	99 (78.6)		282 (79.0)
Cover baby’s body and head				
No	62 (26.8)	26 (20.6)	0.01	88 (24.6)
Yes	169 (73.2)	100 (79.4)		269 (75.4)
Check if the baby is crying				
No	70 (30.3)	37 (29.4)	0.71	107 (30.0)
Yes	161 (69.7)	89 (70.6)		250 (70.0)
Appropriately tie the cord				
No	22 (9.5)	7 (5.6)	0.01	29 (8.1)
Yes	209 (90.5)	119 (94.4)		328 (91.9)
Appropriately cut the cord				
No	31 (13.4)	17 (13.5)	0.97	48 (13.4)
Yes	200 (86.6)	109 (84.5)		309 (86.6)
Advice the mother about umbilical stump care				
No	55 (23.8)	29 (23.0)	0.74	84 (23.5)
Yes	176 (76.2)	97 (77.0)		273 (76.5)
Recorded APGAR score				
No	58 (25.1)	36 (28.6)	0.16	94 (26.3)
Yes	173 (74.9)	90 (71.4)		263 (73.7)
Newborn identification				
No	117 (50.6)	76 (60.3)	0.00	193 (54.1)
Yes	114 (49.4)	50 (39.7)		164 (45.9)
Help baby breastfeed				
No	43 (18.6)	29 (23.0)	0.05	72 (20.2)
Yes	188 (81.4)	97 (77.0)		285 (79.8)
Kept mother and baby together				
No	49 (21.2)	18 (14.3)	0.0	67 (18.8)
Yes	182 (78.8)	108 (85.7)		290 (81.2)
Apply TTC				
No	41 (17.7)	28 (22.2)	0.04	69 (19.4)
Yes	190 (82.3)	98 (77.8)		288 (80.6)
Weighs the baby				
No	21 (9.1)	7 (5.6)	0.02	28 (7.8)
Yes	210 (90.9)	119 (94.4)		329 (92.3)
Advised mother about newborn danger signs				
No	47 (20.3)	25 (19.8)	0.82	72 (20.2)
Yes	184 (79.7)	101 (80.2)		285 (79.8)
Vitamin K given				
No	86 (37.2)	47 (37.3)	0.97	133 (37.3)
Yes	145 (62.8)	79 (62.7)		224 (62.7)
Immunization				
No	125 (54.1)	66 (52.4)	0.57	191 (53.5)
Yes	106 (45.9)	60 (47.6)		166 (46.5)
Recorded the care provided				
No	65 (28.1)	41 (32.5)	0.09	106 (39.7)
Yes	166 (71.9)	85 (67.5)		251 (70.3)
Overall practice				
Poor practice	88 (38.1)	45 (35.7)	0.36	133 (37.3)
Good practice	143 (61.9)	81 (64.3)		224 (62.7)

### Factors affecting the practice of nurses and midwives on immediate newborn care

The result from the chi-square test showed that level of education, work environment and experience, interest in providing newborn care, obtaining newborn care training were significantly associated with immediate newborn care practices of midwives and nurses. Moreover, this analysis found that nurses’ and midwives’ practice on immediate newborn care was significantly associated with their sex and marital status (Table 1).

The multivariable logistic regression analysis revealed that midwives and nurses with inadequate knowledge had lower odds of having a good practice on immediate newborn care [AOR: 0.39; 95% CI (0.25, 0.64)] compared to those having adequate knowledge. Midwives and nurses without interest to provide newborn care were less likely [AOR: 0.35; 95% CI (0.16, 0.74)] to have a good practice on immediate newborn care compared to those who had an interest. In addition, the odds of having a good practical performance on immediate newborn care were lower among midwives and nurses having work experience of lower than six years [AOR: 0.33; 95% CI (0.14, 0.78)] compared to those having six years and above experience. On the other hand, receiving newborn care training [AOR: 0.40; 95% CI (0.23,0.70) and working in hospitals [AOR: 8.56; 95% CI (2.39, 30.63)] were positively associated with good newborn care practices (Table 5).

**Table 5:** Univariable and multivariable logistic regression analysis of Practice of Nurses and Midwives on immediate newborn care in public facilities of Afar Region, Northeastern Ethiopia, 2018

Predictors	Practice		COR (95% CI)	AOR (95% CI)
	Good	Poor		
Knowledge on immediate newborn care				
Inadequate	71 (31.7)	94 (70.7)	0.93 (0.12,0.31)*	0.39 (0.25,0.64)*
Adequate	153 (68.3)	39 (29.3)	1	1
Educational level				
Diploma	122 (54.5)	92 (69.2)	0.53 (0.34,0.84)*	0.78 (0.48,1.26)
Degree	102 (45.5)	41 (30.8)	1	1
Work Experience				
< 5 years	192 (85.7)	122 (91.7)	0.54 (0.26,1.11)	0.33 (0.14,0.78)*
> 6 years	32 (14.3)	11 (8.3)	1	1
Interest in providing newborn care				
No	6 (2.7)	36 (27.1)	0.07 (0.03,0.18)*	0.35 (0.16,0.74)*
Yes	218 (97.3)	97 (72.9)	1	1
Have Work Load				
No	70 (31.3)	45 (33.8)	0.89 (0.56,1.40)	2.09 (1.17,3.73)*
Yes	154 (68.7)	88 (66.2)	1	1
Received newborn care training				
No	119 (53.1)	96 (72.2)	0.44 (0.28,0.69)*	0.40 (0.23,0.70)*
Yes	105 (46.7)	37 (27.8)	1	1
Professional category				
Midwife	143 (63.8)	88 (66.2)	0.90 (0.58,1.42)	0.88 (0.55,1.45)
Nurse	81 (36.2)	45 (33.8)	1	1
Work environment				
Hospital	52 (94.5)	3 (5.5)	13.10 (4.0,48.89)*	8.56 (2.39,30.63)*
Health center	172 (57.0)	130 (43.0)	1	1

*Significant at *p* < 0.05. COR: crude odds ratio; AOR: adjusted odds ratio; CI: confidence interval

## Discussion

This study revealed that the proportion of nurses and midwives having adequate knowledge on immediate newborn care was 53.8%, which is almost similar with the findings in Addis Ababa (51%) [28] and Bahir Dar city (56%) [22]. Differently, this finding is lower than the studies in Jimma (66.4%) [29], eastern Tigray (75%) [21], Northwestern Tigray (64.8%) [30] and Pune city 72% [19]. On the other hand, knowledge of the study participants in this study is slightly higher compared with studies in India (40%) [18] and 41% [17] and Uganda (47%) [20]. This might be due to slight variation in the instrument used and the nature of the study settings. This difference could also be explained by the variation in the type of health professionals included in the study and access to training.

Immediate newborn care practice of respondents in this study was 62.7%, this finding is in line with the finding in Bahir Dar city 59.7% [22] and Northwestern zone of Tigray 59.8% [30]. Our finding in contrary is slightly higher than study in central zone public health facilities of Tigray region 52.4% [31] but lower compared to the finding in Jimma (68.3%) [29], Eastern zone of Tigray (73%) [21], Addis Ababa (81%) [28] and Pune city (98%) [19]. These discrepancies might be due to the difference in the data collection tools and parameter used to measure the respondent’s practice. For instance, a study in central zone of Tigray used the median score as a cutoff point to measure practice.

The result of multivariable logistic regression analysis identified the working environment as a predictor for the knowledge of nurses and midwives on immediate newborn care. Nurses and midwives working in hospitals were nearly 5 times more likely to have adequate knowledge compared to those working in health centers, which is consistent with finding from the studies done in eastern Tigray [21], Bahir Dar city [22] and Afghanistan [8]. However, this finding is different from Uganda, which found no difference in the level of knowledge between two groups [20]. This might be due to the difference in the levels of health care facilities included in the study.

This study also found a significant relationship between knowledge of health professionals and their sex and interest to provide immediate newborn care. Male midwives and nurses had less odds of having adequate knowledge on immediate newborn care compared to females. This finding is inconsistent with the finding in Jimma [29], eastern Tigray [21], Pune city [19] and India [18], which reported an insignificant association between sex of the respondents and their knowledge on newborn care. This variation might be due to the difference in the socio-demographic characteristics of the study participants.

Midwives and nurses without interest to provide immediate newborn care were less likely as compared to those who had the interest to provide. This is almost similar with a finding in Jimma, Ethiopia where health professionals having an interest to work in the delivery room had higher odds of having adequate knowledge comparted to their reference group [29].

Concerning the factors associated with immediate newborn care practice, knowledge on immediate newborn care was found as a predictor of practical performance. The odds of having good practice were 60% lower among nurses and midwives with inadequate knowledge compared to those having adequate knowledge. This is similar to the finding in Jimma, Ethiopia which found a positive statistical relationship between health professional’s knowledge and their practice on newborn care [29]. However, this result is inconsistent with finding in the central zone of Tigray [31] and Afghanistan [8]. This variation might be due to slight difference in the study populations.

Midwives and nurses without interest to provide immediate newborn care were less likely to have a good practice on immediate newborn care compared to those who had an interest. This is similar to the finding reported in Jimma [29].

In addition, the odds of having a good practical performance on immediate newborn care was lower among nurses and midwives having work experience of lower than six years compared to those having six years and above experience. This is different from the studies done in eastern Tigray [21], Addis Ababa [28], Uganda [20] and Afghanistan [8] where there was no significant association between their experience and newborn care practice. This might be due to the difference in the in-service training offered to health care providers.

This study also found that midwives and nurses working at hospitals were nearly nine times more likely to have good newborn care practices compared to those hired in health centers. This finding is similar to a finding reported from central Tigray of Ethiopia [31], where lower odds of good newborn care practices were reported among midwives working in health center.

It was also revealed that nurses and midwives who received newborn care training were more than two times likely to have good newborn care practice compared with those who didn’t receive training, which is consistent with finding in Northwestern Tigray [30] and Afghanistan [8]. In contrary, a cross-sectional study in central Tigray showed that there was no difference between the two groups [31]. This might be due to the difference in the educational background of the study participants.

Furthermore, the odds of good immediate newborn care practice were higher among midwives and nurses who had workload compared to those without workload. This association was not significant in Northwestern Tigray of Ethiopia [30]. This might be due to difference the number of health professionals in the study areas.

### Strength and limitations

Use of direct observation to assess the practical performance of midwives and nurses is the major strength of this study. However, there might be observation bias and the cross-sectional nature of the study does not confirm the cause and effect relationship between predictors and outcome variables.

### Conclusions

This study showed that a significant number of nurses and midwives had inadequate knowledge and poor practice on immediate newborn care. Work environment, sex and interest in providing newborn care were statistically significant predictors for knowledge on newborn care. Moreover, it was revealed that the practice of midwives and nurses on newborn care was influenced by their knowledge, work experience, interest in providing the care, receiving newborn care training, workload and environment.

Based on these findings, priorities have to be given to create experience sharing opportunities between midwives and nurses working in hospitals and health centers. Providers’ interest should be considered as the part of the criteria to assign midwives and nurses to the newborn care unit. In addition, strengthening provision of refreshment training on regular basis coupled with strict follow up is very crucial to improve their knowledge and skills on newborn care.

## Data Availability

The datasets used and/or analyzed during the current study are available from the corresponding author on reasonable request. All coauthors gave full responsibility to the corresponding author to share and/or discuss with editors and reviewers.

## Abbreviations

AOR: Adjusted odds ratio
CI: Confidence interval
COR: Crude odds ratio
EDHS: Ethiopia Demographic and Health Survey
RERC: Research Ethics Review Committee
WHO: World Health Organization
